# Metabolic map of Brucellosis: From acute and chronic phase adaptation to targeted nanodrug design

**DOI:** 10.1016/j.mtbio.2025.102684

**Published:** 2025-12-19

**Authors:** Lan Yu, Manyi Wen, Haitao Ding

**Affiliations:** a Inner Mongolia Academy of Medical Sciences, Inner Mongolia People's Hospital, Hohhot, Inner Mongolia Autonomous Region, China; b Key Laboratory of Metabolic Disease Regulation and Biomedical Therapy of Inner Mongolia Autonomous Region, China; c Clinical Medical Laboratory Center, Inner Mongolia People's Hospital, Hohhot, Inner Mongolia Autonomous Region, China

**Keywords:** Brucellosis, Metabolic adaptation, Host-pathogen interaction, Nanotechnology, Drug delivery, Intracellular persistence

## Abstract

Brucellosis is a globally prevalent zoonotic disease caused by *Brucella* species, leading to significant public health and economic burdens, especially in developing regions. As a facultative intracellular pathogen, *Brucella* establishes long-term persistence within host cells, often resulting in chronic infections that are difficult to eradicate. The metabolic interplay between the host and the pathogen plays a crucial role in disease progression, influencing both immune evasion and bacterial survival. This review systematically outlines the metabolic adaptations of *Brucella* during acute and chronic phases of infection, covering central carbon metabolism, nitrogen utilization, lipid and nucleotide turnover, and metal homeostasis. We further discuss how these metabolic insights can inform the rational design of nanoparticle-based drug delivery systems for targeted antimicrobial therapy. By integrating host and bacterial metabolic perspectives, this work aims to provide a comprehensive framework for understanding brucellosis pathogenesis and to inspire novel diagnostic and therapeutic strategies.

## Graphical abstract

**Figure:**
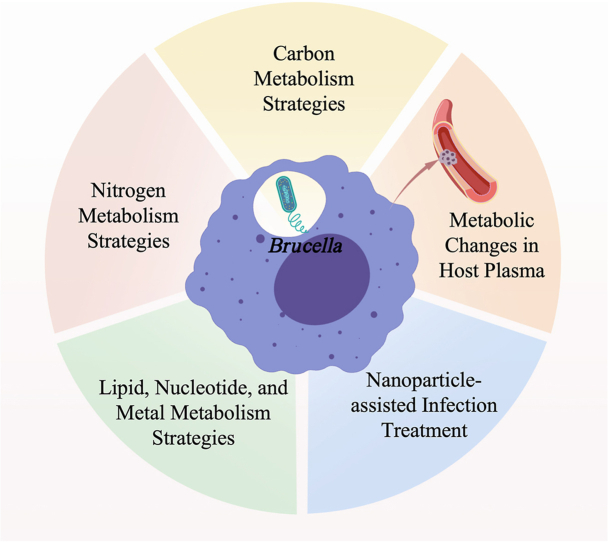

## 1 Introduction

Brucellosis, an important zoonotic infectious disease caused by *Brucella* spp., is prevalent globally, particularly in developing countries, inflicting severe economic losses on public health and animal husbandry. As facultative intracellular pathogenic bacteria, the pathogenicity of *Brucella* primarily relies on its capacity to invade host cells, where it survives and replicates [[1], [2], [3], [4]]. Following infection, *Brucella* typically establishes persistent infection, leading to chronic brucellosis, for which there is currently no radical cure [5].

The infection process of *Brucella* is generally divided into acute phase and chronic phase. The acute phase is characterized by non-specific symptoms such as fever, fatigue, and joint pain, during which the bacteria proliferate rapidly and spread within the host. Without timely and effective clearance, the infection progresses to the chronic phase, manifesting as recurrent symptoms, formation of local lesions, and persistent infection that is difficult to eliminate completely. *Brucella* succeeds in establishing chronic infection through a series of "stealth" strategies, including evasion of the host's innate and adaptive immune responses, regulation of cellular processes such as autophagy and apoptosis, and potentially involvement of small non-coding RNAs [5]. Host cells, especially immune cells like macrophages, undergo significant metabolic reprogramming during infection—this serves as both a host strategy to combat pathogens and a potential target for pathogen exploitation [6].

As an intracellular pathogen, the survival and replication of *Brucella* are closely linked to the metabolic state of host cells [4]. During different infection phases, the intracellular microenvironment of *Brucella* (e.g., from early phagosomes to endoplasmic reticulum-derived replicative vacuoles) undergoes dynamic changes in pH, oxygen levels, and the type and concentration of nutrients [3]. *Brucella* must possess flexible metabolic regulation capabilities to efficiently acquire energy and biosynthetic precursors under these variable conditions, sustaining its survival, replication, and evasion of host clearance [[7], [8], [9]]. For instance, the Type IV secretion system (VirB system) of *Brucella* is a key virulence factor that secretes effector proteins to hijack host cell pathways, promoting bacterial replication within host cells and inducing persistent infection—this undoubtedly requires metabolic support from the bacteria in terms of energy and substance metabolism [10].

*Brucella* employs dynamic metabolic adaptations to thrive in hostile host environments—especially within macrophages during both acute and chronic infection [11]. Its ability to utilize alternative carbon sources and sustain a low-pH-tolerant state contributes significantly to intracellular persistence and immune evasion. Conventional antibiotics often fail to eradicate the pathogen due to poor cellular uptake, acidic degradation, and enzymatic inactivation [12,13]. Nanomaterial-based drug delivery systems present a promising strategy to overcome these limitations [14,15]. By encapsulating antibiotics within pH-sensitive nanoparticles, therapeutic agents can be efficiently delivered to infection sites, minimizing degradation and enhancing intracellular accumulation. Such systems not only improve drug stability and bioavailability but also may reduce treatment duration and mitigate relapse. This approach represents a novel therapeutic paradigm for combating *Brucella* infections.

This review aims to integrate existing research findings and systematically explore the metabolic changes induced by *Brucella* during the acute and chronic phases. Starting from the macroscopic metabolic changes in host plasma, we analyze the metabolic characteristics of human and mouse hosts at different infection stages. Subsequently, we delve into the similarities and differences in *Brucella*'s own central carbon metabolism (CCM), nitrogen metabolism, metabolism of other energy substances (including lipids, nucleotides, and metals), and stringent response-associated metabolic pathways between these two infection phases. By comparing these metabolic changes and reviewing the nanoparticle-assisted drug delivery systems for *Brucella* infection treatment, we hope to gain a more comprehensive understanding of the pathogenic mechanisms and persistent infection strategies of *Brucella*, thereby providing new insights and targets for the diagnosis, treatment, and vaccine development of brucellosis. Early metabolic studies already suggested that *Brucella* possesses an unusually streamlined CCM and relies on erythritol, amino acids, and TCA cycle intermediates for replication, laying the foundation for later omics-driven metabolic frameworks [16,17].

## 2 Metabolic alterations during *Brucella* infection

*Brucella* infection triggers profound metabolic perturbations at the systemic and cellular levels, reflecting the ongoing bidirectional interaction between host immunity and microbial survival strategies. These changes evolve across acute and chronic stages and involve both host-driven metabolic reprogramming and *Brucella*-centered adaptive metabolism. To improve conceptual clarity, the section is subdivided into two components while maintaining the mechanistic continuity required to interpret host-pathogen metabolic interplay.

### 2.1 Host metabolic reprogramming during infection

Host metabolism undergoes dynamic remodeling during *Brucella* infection, driven by inflammation, immune activation, mitochondrial stress, and altered organ function. Plasma metabolomics from clinical cohorts have shown extensive perturbations in amino acid, purine, bile acid, lipid, and central energy pathways during acute infection [[18], [19], [20]] (Table 1). These signatures reflect not only immunological activation but also the systemic consequences of *Brucella* persistence and intracellular stress responses.

**Table 1:** Summary of differential metabolites.

Metabolites	Change Direction	Metabolic Pathway	References
**Taurocholic acid**	Up ↑	Primary bile acid biosynthesis	Niu et al. [18]
**cis-4-hydroxy-D-proline**	Down ↓	D-amino acid metabolism	Niu et al. [18]
**Inosine**	Down ↓	Purine metabolism	Niu et al. [18]
**Hypoxanthine**	Down ↓	Purine metabolism	Niu et al. [18]
**L-Iditol**	Up ↑	Fructose and mannose metabolism	Niu et al. [18]
**Taurochenodeoxycholic acid, TCDCA**	Up ↑	Primary bile acid biosynthesis	Niu et al. [18]
**Glycocholic acid, GCA**	Up ↑	Primary bile acid biosynthesis	Niu et al. [18]
**Azelaic acid, AZA**	Down ↓	Fatty acid biosynthesis/degradation	Niu et al. [18]
**Glycoursodeoxycholic acid, GUDCA**	Up ↑	Secondary bile acid biosynthesis	Niu et al. [18]
**L-Kynurenine**	Up ↑	Tryptophan metabolism	Li et al. [19]
**(3,4-Dimethoxyphenyl)acetic acid**	Up ↑	Phenylalanine metabolism	Li et al. [19]
**D-Sphingosine**	Up ↑	Sphingolipid metabolism	Li et al. [19]
**D-(+)-Proline**	Up ↑	Arginine & proline metabolism	Li et al. [19]
**2-Amino-1,3-octadecanediol**	Up ↑	Sphingolipid metabolism	Li et al. [19]
**Kahweol**	Down ↓	Diterpenoid biosynthesis	Li et al. [19]
**2-Hydroxycinnamic acid**	Up ↑	Phenylpropanoid biosynthesis	Li et al. [19]
**Kynurenic acid**	Up ↑	Tryptophan metabolism	Li et al. [19]
**5-(tert-butyl)-2-methyl-N-(4-nitrophenyl)-3-furamide**	Up ↑	Xenobiotics biodegradation	Li et al. [19]
**2-chloro-6-(4-methoxyphenoxy)benzonitrile**	Up ↑	Xenobiotics biodegradation	Li et al. [19]
**1,4-dihydroxyheptadec-16-en-2-yl acetate**	Up ↑	Lipid metabolism	Li et al. [19]
**Lignoceric acid**	Down ↓	Fatty acid elongation	Li et al. [19]
**Pentacosanoic acid**	Down ↓	Fatty acid elongation	Li et al. [19]
**Xanthine**	Up ↑	Purine metabolism	Li et al. [19]
**L-Phenylalanine**	Up ↑	Phenylalanine metabolism	Li et al. [19]
**D-(+)-Tryptophan**	Up ↑	Tryptophan metabolism	Li et al. [19]
**Oleoyl-L-α-lysophosphatidic acid**	Down ↓	Glycerophospholipid metabolism	Li et al. [19]
**γ-Aminobutyric acid (GABA)**	Up ↑	Alanine, aspartate & glutamate metabolism	Li et al. [19]
**L-Glutamic acid**	Up ↑	Alanine, aspartate & glutamate metabolism	Li et al. [19]
**Citric acid**	Down ↓	TCA cycle	Li et al. [19]
**2-(1H-benzimidazol-2-yl)-3-(1,3-benzodioxol-5-yl)acrylonitrile**	Up ↑	Xenobiotics biodegradation	Li et al. [19]
**Perfluorooctanoic acid**	Up ↑	Xenobiotics biodegradation	Li et al. [19]
**4-Hexylresorcinol**	Down ↓	Xenobiotics biodegradation	Li et al. [19]
**Sorbitan monopalmitate**	Up ↑	Glycerolipid metabolism	Li et al. [19]
**Deoxycholic acid**	Up ↑	Secondary bile acid biosynthesis	Li et al. [19]

In the acute phase, differential enrichment of biomarkers such as taurocholic acid, glycocholic acid, and taurochenodeoxycholic acid suggests hepatic metabolic involvement and immunomodulatory bile acid signaling [18]. Meanwhile, altered inosine, hypoxanthine, and xanthine levels indicate increased nucleotide turnover, immune cell activation, and purine recycling [18,19]. Amino acid pathway disruptions—including elevated kynurenine and phenylalanine—further support immune activation-associated tryptophan metabolism and systemic inflammatory compensation [19].

**Fig. 1:**
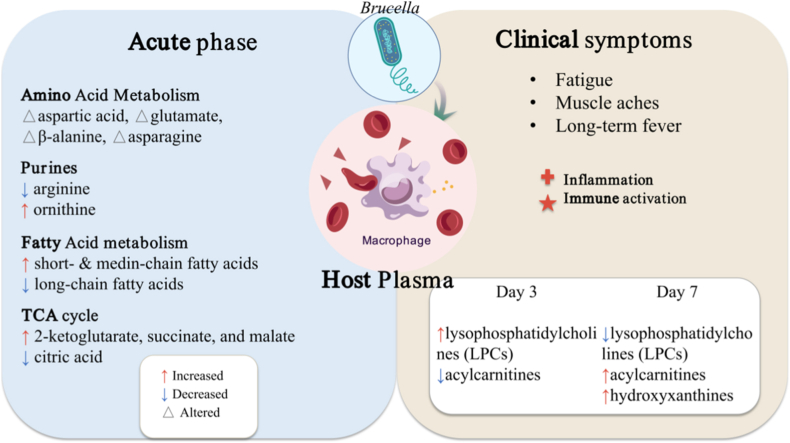
Host Metabolic Reprogramming During *Brucella* Infection. Clinical patients with acute brucellosis exhibit fatigue, myalgia, and persistent fever, accompanied by host inflammatory and immune activation. Plasma metabolomics reveal widespread alterations across multiple pathways (left panel). In mice, metabolite profiles differ between early (3 days) and later (7 days) infection stages (bottom right panel).

Energy metabolism is also markedly affected. Targeted metabolomics revealed reduced citrate but accumulation of succinate, lactate, 2-oxoglutarate, and malate, consistent with impaired TCA cycle flux and enhanced aerobic glycolysis (Table 2), a pattern classically associated with M1-like macrophage immunometabolism [20,21]. The parallel disruption of lipid metabolism, particularly characterized by a reduction in long-chain fatty acids alongside an increase in short- and medium-chain lipid intermediates, indicates a metabolic shift toward fatty acid mobilization and mitochondrial stress responses [20]. Comparable trends are documented in murine infection models, where early infection (3 dpi) is characterized by increased lysophosphatidylcholines, while late infection (14 dpi) shows accumulation of acylcarnitines and hydroxypurines, reflecting stage-dependent lipid remodeling and immune-metabolic compensation [22] (Fig. 1).

**Table 2:** Summary of differential metabolites in Fu et al. [20].

Metabolic Pathway	Counts	Change Direction	Example metabolites	References
**Long-chain unsaturated fatty acid metabolism**	11	Down ↓	Oleic acid, Linoleic acid	Fu et al. [20]
**Acylcarnitine metabolism**	6	Down ↓	Acetylcarnitine, Decanoylcarnitine	Fu et al. [20]
**Amino acid metabolism**	5	Down ↓	β-Alanine, Glutamine	Fu et al. [20]
**Amino acid metabolism/Urea cycle**	6	Up ↑	Glutamate, Ornithine	Fu et al. [20]
**Medium-chain fatty acid metabolism**	16	Up ↑	Suberic acid, Sebacic acid	Fu et al. [20]
**Short-chain fatty acid metabolism**	10	Up ↑	Acetic acid, Propanoic acid	Fu et al. [20]
**Organic acid metabolism & TCA intermediates**	14	Up ↑	Succinic acid, Lactic acid	Fu et al. [20]
**Carbohydrate metabolism**	4	Up ↑	Fructose, Xylulose	Fu et al. [20]
**Other/unspecified**	11	Down ↓	–	Fu et al. [20]

### 2.2 Brucella metabolic adaptation to the host microenvironment

In parallel with host metabolic remodeling, *Brucella* implements flexible metabolic responses that allow survival in fluctuating nutritional and immunological contexts. The pathogen's metabolic strategy is phase-dependent: during acute infection, rapid intracellular replication favors metabolic pathways capable of efficiently leveraging host-derived carbohydrates, amino acids, and nucleotide intermediates, whereas chronic infection promotes energy-efficient maintenance programs aligned with persistence.

Consistent with metabolomic evidence of elevated host purine and amino acid turnover, *Brucella* utilizes high-capacity uptake and salvage pathways to access these nutrients [9,23]. The pathogen's distinctive metabolic architecture—characterized by the absence of canonical glycolysis and reliance on the pentose phosphate pathway, erythritol metabolism, and gluconeogenesis—aligns with substrate availability in infected macrophages and reproductive tissues [8,24]. As infection progresses and nutrient resources decline, *Brucella* transitions toward metabolic states dominated by gluconeogenesis, β-oxidation, and stringent-response-regulated biomass restriction, supporting long-term intracellular persistence ([2,5]; X et al., 2024).

These metabolic adaptations are coordinated by global regulatory systems, including BvrR/BvrS, NtrY/NtrX, and the stringent response regulator Rsh, which integrate metabolic status with virulence expression, oxidative stress defense, and replicative control [[25], [26], [27]]. This enables *Brucella* to maintain a low-replication "stealth" phenotype capable of resisting immune clearance while preserving metabolic flexibility.

Overall, plasma metabolomics indicate broad host reprogramming during *Brucella* infection. Early disease emphasizes inflammation-linked pathways (amino acids, purines), whereas progression involves deeper disruption of energy (glycolysis/TCA, fatty acids) and amino acid/urea metabolism. These shifts reshape nutrient availability for intracellular *Brucella*, prompting metabolic adaptation; accelerated glycolysis with impaired TCA likely alters intermediate pools, creating distinct intracellular microenvironments [6,20,21]. In macrophages, reduced oxidative phosphorylation and increased glycolysis associate with HIF-1α stabilization via STING and IRE1α, reinforcing pro-inflammatory programs while modifying the niche. Iron handling is also temporal: macrophage ferritin and transferrin are suppressed early but may rise later, directly affecting intracellular iron, a key growth-limiting factor ([6,20,21], Fig. 1). Together, host metabolic rewiring and *Brucella* adaptive metabolism define a reciprocal metabolic interface that evolves across infection stages. Host immunity reshapes nutrient pools and cellular microenvironments, while *Brucella* exploits and adapts to these constraints through specialized metabolic programs. Understanding this bidirectional metabolic exchange provides a mechanistic basis for biomarker development and therapeutic innovation, including precision antimicrobial strategies and metabolically informed nanotherapeutic delivery systems.

## 3 CCM strategies of *Brucella*

**Fig. 2:**
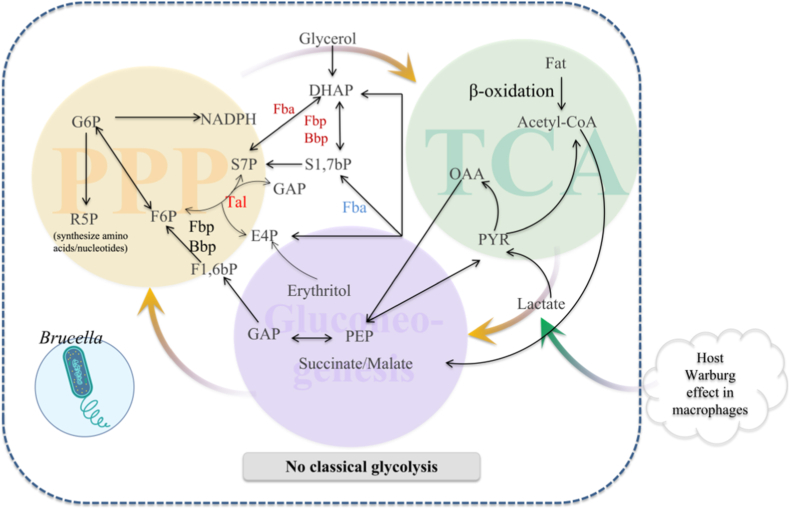
CCM of *Brucella*. *Brucella* lacks classical glycolysis (center) and instead utilizes the pentose phosphate pathway (left), gluconeogenesis (center, purple), and fatty acid β-oxidation feeding the TCA cycle (right, green). Glycerol and erythritol (center) serve as key carbon sources, while succinate and malate act as central intermediates. Host macrophages (lower right) undergo a Warburg-like shift, generating lactate that further supports bacterial metabolism. (For interpretation of the references to colour in this figure legend, the reader is referred to the Web version of this article.)

CCM in *Brucella* shows distinctive features [28]. All species depend on a complete pentose phosphate pathway (PPP) for hexose, pentose, and erythritol catabolism/anabolism, and PPP is the main route for hexose breakdown in classical lineages [8]. Some rodent-associated strains retain an ED pathway, likely an ancestral trait. *Brucella* lacks key enzymes of classical glycolysis (e.g., phosphofructokinase, phosphoenolpyruvate synthase), confirming the absence of the canonical route [8]. When carbohydrates are limited, gluconeogenesis (via PpdK and Mae) supplies TCA intermediates and supports virulence. Genomes encode complete TCA and glyoxylate cycles [29], enabling use of C2 substrates (e.g., acetyl-CoA) and gluconeogenic growth (Fig. 2). These pathway characteristics are consistent with earlier biochemical work demonstrating the absence of classical phosphofructokinase activity and reliance on alternative hexose processing pathways, long before genome-scale analyses confirmed the lack of canonical glycolysis in *Brucella* [30,31].

Carbon use is tissue- and phase-dependent. Early after entry and rBCV formation [3,10], erythritol-rich sites such as placenta provide abundant substrate, driving rapid replication. Lázaro-Antón et al. [24] uncovered a gluconeogenic route that channels erythritol-derived erythrose-4-phosphate into PPP via Fba and a broad-spectrum bisphosphatase (Bbp), generating fructose-6-phosphate and sedoheptulose-7-phosphate. This linkage between erythritol use and PPP explains high bacterial burdens in reproductive tissues and the association with abortion and infectivity (Fig. 2).

In macrophages, *Brucella* likely taps glucose, amino acids, and other small carbon sources [8,23]. Host cells undergo early immunometabolic reprogramming (↑glycolysis, ↓oxidative phosphorylation) [6,21], and vaccine-induced antibodies can constrain infection by limiting host glucose [32]. In THP-1 cells, infection reduces amino acid metabolism but increases glucose consumption and lactate release, an aerobic glycolysis ("Warburg-like") shift also triggered by heat-killed bacteria ([33], Fig. 2), allowing *Brucella* to exploit host carbon without compromising cell viability.

With progression to chronic infection, the niche becomes nutrient-poor and hostile, favoring a "stealth" strategy with low-level replication [5]. Profound CCM rewiring is implicated [2,34,35]: *Brucella* likely leans on gluconeogenesis and the glyoxylate cycle, using non-carbohydrate substrates (amino acids, lactate, glycerophosphate) to sustain biomass and maintenance [2,24]. Analogous adaptations—upregulating gluconeogenesis and arginine deiminase under starvation—have been observed in chronic osteomyelitis by other pathogens [34]. Metabolic flexibility across carbon sources underpins persistence [8].

Across phases, PPP and TCA provide energy and precursors, but substrate preference shifts. Acute infection favors readily available substrates (notably erythritol in specific tissues) alongside host-derived hexoses, pentoses, and amino acids [8,24]. Chronic infection relies more on gluconeogenesis (e.g., amino acids, lactate), with an increased role for the glyoxylate cycle ([34], Fig. 2).

CCM is tightly regulated. The BvrR/BvrS two-component system directly modulates genes for PPP and gluconeogenesis and is linked to intracellular survival [8,25], permitting dynamic redirection of carbon flux: toward biomass/energy during nutrient-replete acute infection, and toward maintenance/stress responses under chronic limitation.

## 4 Nitrogen Metabolism Strategies of *Brucella*

Nitrogen sources—ammonia, amino acids, peptides, and polyamines—are essential for *Brucella* growth, and their acquisition and processing are staged [9]. *Brucella* is an amino acid autotroph, capable of synthesizing all essential nutrients from simple nitrogen sources such as ammonia; it grows in vitro on ammonia or glutamate as its sole carbon/nitrogen source [9]. Notably, the *Brucella* genome encodes more than twice the number of amino acid/peptide/polyamine transporters compared to amino acid heterotrophic intracellular bacteria such as *Legionella pneumophila*, implying a strong dependence on host-derived amino acids/peptides as a nitrogen (and sometimes carbon) source within the vacuolar microenvironment.

Nitrogen metabolism is linked to virulence: mutations in multiple amino acid biosynthetic pathways can attenuate infection [9,36], highlighting the importance of autotrophy when host supply is limited or biased.

**Fig. 3:**
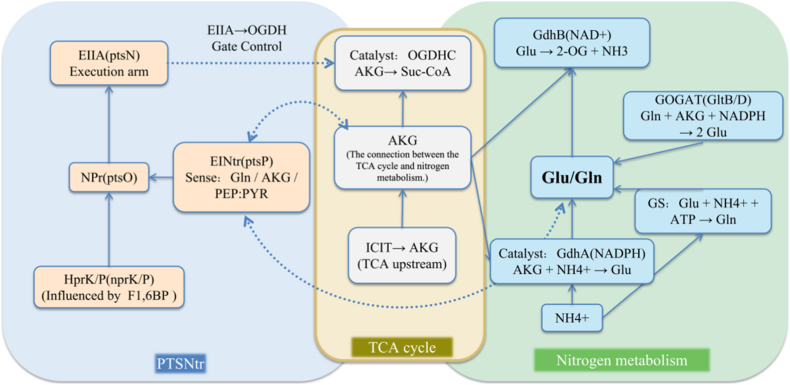
Nitrogen Metabolism Strategies of *Brucella*. The PTSNtr system (left, blue) integrates carbon and nitrogen signals through HprK/P, NPr, EINtr, and EIIA, regulated by F1,6BP and PEP/PYR. The TCA cycle (center, yellow) provides intermediates such as α-ketoglutarate (AKG) linking carbon flux to nitrogen assimilation. Nitrogen metabolism (right, green) involves glutamate/glutamine cycling, where GDH, GOGAT, and GS catalyze the interconversion of NH4+, Glu, and Gln, coordinated with TCA intermediates. Together, these pathways connect carbon metabolism, nitrogen utilization, and energy balance during bacterial growth. (For interpretation of the references to colour in this figure legend, the reader is referred to the Web version of this article.)

In acute infection, rapid replication requires abundant nitrogen. *Brucella* preferentially utilizes amino acids and polyamines, activates denitrifying respiration under hypoxic conditions, and uptakes nitrogen through the GS-GOGAT network, regulated by the NtrYX-Rsh network—factors that collectively support its intracellular survival while maintaining its "stealth" state [37,38]. A rich arsenal of transporters facilitates uptake from the cytosol/vacuole [9]. Host plasma metabolomics reveals alterations in the amino acid pool (e.g., glutamate, aspartate, and asparagine) during acute brucellosis, reflecting both host responses and reshaping bacterial substrate availability ([19,20], Fig. 3).

With the transition to chronic infection, *Brucella* adopts a program of sustained survival under more stringent nutrient limitation, including nitrogen. Their strategy emphasizes "nitrogen resource conservation"—prioritizing amino acid synthesis while suppressing catabolism [39]; utilizing nutrients provided by M2-like macrophages [40]; downregulating costly processes such as denitrification and the urea cycle [39,41]; and relying on key substrates (such as arginine) to maintain persistence. Glutamate can serve as both a carbon and nitrogen source, and denitrification may become more important in chronic, hypoxic niches. Consistent with the reduced activity of persisters (X et al., 2024), nitrogen demand decreases but does not disappear; a supply is still required for maintenance and repair. Upregulation of the arginine deiminase pathway in chronic *Staphylococcus aureus* osteomyelitis illustrates how amino acid catabolism can provide ATP during starvation [34]; although direct evidence is lacking in *Brucella*, its utilization of glutamate suggests this possibility [9].

Regulation is multifactorial. BvrR/S directly controls nitrogen-related genes, orchestrating the carbon-nitrogen program for intracellular adaptation [25]; proteomic studies under simulated intracellular stress conditions have demonstrated remodeling of nitrogen proteins [29]. The two-component systems NtrY/NtrX and PrrBA integrate redox and oxygen signaling: under hypoxic conditions, NtrY activates NtrX to induce denitrification genes, while PrrBA collaborates with NtrY/NtrX to regulate denitrification and high-affinity cytochrome oxidase [26,42]. The PTSNtr pathway further connects carbon and nitrogen status; mutations (e.g., EINtr, BMEI0190) reduce virulence in cellular models, highlighting its role in acute phase control ([43], Fig. 3).

## 5 Metabolic strategies of *Brucella* for other energy substances

Beyond CCM and nitrogen metabolism, *Brucella* survival and pathogenicity also rely on lipid, nucleotide, and metal metabolism, with acquisition, synthesis, and utilization strategies that likely diverge across acute and chronic phases [9].

### 5.1 Lipid metabolism

Lipids underpin membrane architecture, energy storage, and signaling, and *Brucella* lipid flux is integral to membrane integrity, intracellular survival, and host interaction. In acute infection, rapid division increases lipid demand; the pathogen can draw on host fatty acids/precursors and channel them into fatty acid synthesis. Host lipids are broadly mobilized during acute inflammation—e.g., in LPS-treated rats, lipids were the major discriminants of survival [44]—potentially creating opportunities for bacterial lipid acquisition. *Brucella* surface lipids (notably LPS) are key virulence determinants and require sufficient precursors. In chronic infection, lipid use is likely reprogrammed toward persistence: β-oxidation of host fatty acids may supplement energy under carbon limitation, while remodeling host lipid pathways helps establish a supportive niche. Adjustments in membrane lipid saturation/head-group composition can enhance stability under acidity, oxidative stress, and antimicrobial peptides. Lipid metabolism interfaces with virulence systems (e.g., VirB) that govern BCV biogenesis and function; fine control of lipid flux likely sustains BCV integrity and long-term intracellular survival ([45], Table 3).

**Table 3:** Comparison of metabolic strategies of *Brucella* for other energy substances.

Pathway	Acute-phasestrategy	Chronic-phasestrategy	Commonalities	References
Lipid	Demand rises with rapid replication; leverages host fatty acids/precursors to fuel lipid synthesis; reinforces LPS biosynthesis; exploits host lipid mobilization during acute inflammation	Shifts toward host lipid use (β-oxidation) under carbon limitation; remodels host lipid pathways to build a permissive niche; tunes membrane acyl saturation/headgroups to tolerate acidity/oxidative stress/AMPs; VirB-linked control preserves BCV integrity/function	Lipids underpin membranes, energy, and signaling; tightly coupled to virulence; host–pathogen interactions co-shape the lipid microenvironment	[44,45]
Nucleotide	High replication elevates demand; uses de novo plus salvage of host precursors; may tap/perturb host folate/one-carbon pathways (analogy to viral hijacking)	Favors energy-efficient salvage under nutrient stress; integrates with the stringent response; ppGpp adjusts rRNA and nucleotide biosynthesis; potential interference with host nucleotide metabolism impacts immune cell function	Dynamic de novo–salvage balance to match growth and energy constraints; bidirectional cross-talk with host metabolism and immunity	[27,46,47]
Metal	Rapid acquisition of essential metals; Fe via siderophores or host transferrin/lactoferrin; QS coordinates C/N metabolism to optimize siderophore production and sustain growth; Mn-dependent SodA counters oxidative stress	Adapts to scarcity/toxicity imposed by nutritional immunity; enhances scavenging from host pools and efflux/detox (Cu, Zn); reorganizes energy metabolism; Fe levels modulate virulence gene expression to support persistence	Precise metal homeostasis balances cofactor needs vs toxicity; tightly integrated with CCM, virulence regulation, and energy metabolism	[18,29,48,49]

### 5.2 Nucleotide metabolism

Nucleotides are required for genome maintenance, translation capacity, and energy/signaling (ATP, cAMP). During acute infection, heightened replication drives increased nucleotide demand; *Brucella* can engage de novo synthesis and salvage host-derived precursors. Analogous to SARS-CoV-2 hijacking folate/one-carbon pathways [46,47], *Brucella* may co-opt host nucleotide biosynthesis. In chronic infection, metabolism adapts to low-level replication and nutrient stress: salvage pathways become more prominent, and the stringent response (ppGpp) modulates rRNA and nucleotide biosynthesis to conserve resources [27]. Cross-talk with host immunity is probable: interference with host nucleotide pools (e.g., via serine/one-carbon flux) may dampen immune cell proliferation, whereas host restriction of nucleotide availability may curb persistence (Table 3).

### 5.3 Metal metabolism

Iron, zinc, manganese, and copper serve as cofactors and redox mediators but can be toxic in excess; hosts deploy "nutritional immunity" to restrict essential metals or load toxic ones, compelling *Brucella* to tightly control metal homeostasis. In acute infection, efficient uptake supports rapid growth. For iron, pathogens deploy siderophores or tap host carriers (lactoferrin, transferrin); quorum sensing can tune siderophore output by integrating carbon-nitrogen status to secure iron for fast growth [18], underscoring links to central metabolism. Mn-dependent SodA is critical for oxidative stress defense and early infection [48]. In chronic infection, strategies shift toward heightened adaptability: optimization of uptake and use of host metal pools under scarcity, balanced by efflux/detoxification systems to mitigate copper/zinc toxicity [49], often coupled to broader energy-metabolic reorganization. Metal availability also regulates virulence gene expression, suggesting that precise metal control sustains virulence functions and persistence in the intracellular niche. Proteomics under simulated intracellular stress revealed altered abundance of OXPHOS and TCA proteins [29], many of which are metalloenzymes, indirectly highlighting the centrality of metal metabolism to intracellular adaptation (Table 3).

Building upon the above mechanistic framework, infection-stage-specific metal acquisition strategies further refine *Brucella*'s metabolic adaptation and persistence program, particularly under host-driven metal starvation. Beyond the acute infection stage, *Brucella* must adapt to a progressively metal-depleted intracellular environment caused by host nutritional immunity. While iron acquisition and SodA-mediated oxidative defense dominate early-stage survival, chronic persistence relies on more specialized and energy-efficient metal management strategies [9,48]. During chronic infection, *Brucella* upregulates high-affinity ABC-type transporters and metal-specific permeases to access trace levels of Mn, Zn, and Fe, reflecting a shift toward competitive micronutrient scavenging under metal restriction imposed by host NRAMP1, calprotectin, and lactoferrin responses [29,50].

Notably, manganese appears increasingly important during persistence, functioning not only as a cofactor for SodA and ribonucleotide reductases but also as a substitute catalytic metal for enzymes normally dependent on Fe under oxidative or hypoxic restriction [42,51]. Zinc buffering pathways, including Zur-regulated genes, further contribute to metabolic quiescence and intracellular survival. These adaptations align with *Brucella*'s denitrification capacity and its ability to respire under low-oxygen, metal-limited niches characteristic of chronic infection [2,42].

These mechanistic insights also highlight metal metabolism as a promising therapeutic axis. Potential strategies include metal chelation, competitive metal acquisition blockade, or the development of nano-formulated delivery systems capable of disrupting metal gradients essential for persistent intracellular survival. Recent work on transition metal-based nanozymes demonstrates that mitochondria-targeted copper/iron platforms can reprogram redox homeostasis and metal ion balance to induce ferroptosis and cuproptosis in diseased cells, underscoring the potential of metabolism-informed metal disruption strategies [52]. In parallel, silver nanoparticle formulations have been shown to efficiently enter host cells, suppress intracellular Salmonella Typhimurium infection, and activate dendritic cells, thereby enhancing antimicrobial immune responses [53]. Together, these nano-enabled metal-targeting approaches suggest that analogous metabolism-informed metal disruption could be feasible in *Brucella* as well.

## 6 Nanoparticle-assisted drug delivery for Brucella infection treatment

**Fig. 4:**
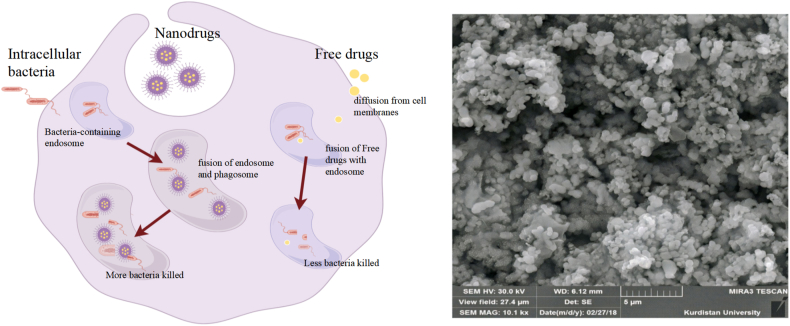
Left: Comparison of free drug with nanodrug in reducing the number of intracellular bacteria adapted from Ref. [14]. Copyright 2022, Elsevier. Right: Field emission scanning electronic microscope images of DOX-SLN (Hosseini et al. Antimicrobial Resistance and Infection Control (2019)), licensed under [CC BY 4.0].

A growing understanding of *Brucella*'s stage-specific metabolic adaptations is refining our conceptual framework of its persistence and provides a mechanistically grounded rationale for designing precision nanotherapeutics. This pathogen exhibits remarkable metabolic plasticity, rewiring its central carbon and energy metabolism across the acute-chronic infection continuum [54]. Rather than treating nanotechnology as an isolated antimicrobial platform, connecting it to these metabolic vulnerabilities enables a shift from empirical drug delivery toward targeted metabolic intervention. Such a metabolism-informed nanodrug strategy has the potential to disrupt the intracellular lifestyle of *Brucella* more efficiently, particularly given the dynamic metabolic rewiring that accompanies the transition from the acute proliferative phase to the chronic persistence phase. To visually summarize this translational concept, the left panel of Fig. 4 illustrates the metabolic rewiring trajectory of *Brucella* across acute and chronic infection (adapted from Ref. [1]), whereas the right panel presents a representative nanodelivery proof-of-concept using doxycycline-loaded solid lipid nanoparticles targeting intracellular *Brucella* melitensis within macrophages [11], together demonstrating how mechanistic metabolic insight can inform rational nano-formulation and targeted antimicrobial strategies.

**Fig. 5:**
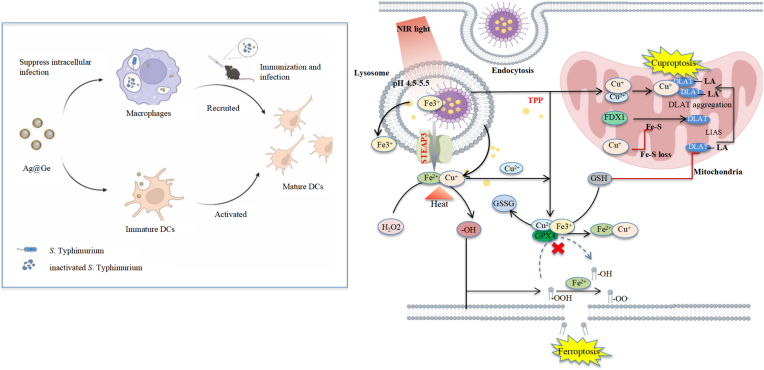
Left: Schematic illustration of the antibacterial and immune adjuvant effects of Ganoderma extract-capped silver nanoparticles (Ag@Ge) against intracellular Salmonella Typhimurium. Adapted from Ref. [53]. Copyright 2022, Elsevier.

The development of nanocarrier-assisted antibiotic delivery for brucellosis demonstrates a clear evolutionary path from foundational concepts to sophisticated, metabolism-informed strategies. Initial proof-of-concept was established with doxycycline-loaded solid lipid nanoparticles (SLNs), which showcased superior physicochemical stability, sustained drug release, and enhanced intracellular delivery compared to the free antibiotic [14]. Building on this platform, therapeutic refinement advanced towards co-delivery, exemplified by formulations combining doxycycline with hydroxychloroquine to modulate the acidic phagolysosomal environment, which demonstrated increased antibacterial activity across infection models (Moez et al., 2023). A similar co-delivery system with streptomycin and hydroxychloroquine further achieved improved bacterial clearance and began to address host pathophysiology by partially restoring dysregulated metal ion balance and inflammatory markers (Karimitabar et al., 2023). Concurrently, the use of quantum-dot-labeled SLNs provided crucial visual confirmation of nanoparticle internalization and arrival at the intracellular niche of *Brucella* (Karimitabar et al., 2023), validating the targeting premise. This entire approach is fundamentally guided by the pivotal, stage-specific metabolic vulnerabilities of *Brucella*. During acute infection, characterized by elevated pentose phosphate pathway activity and oxidative stress dependence, nanocarriers can deliver agents to amplify metabolic stress, as supported by the enhanced efficacy of doxycycline-loaded SLNs in restoring trace element homeostasis [11]. In contrast, for the hypoxic, metal-dependent chronic phase, strategies like pH-responsive hydrogels incorporating drug-loaded nanoparticles effectively leverage the infection microenvironment for targeted release (Abo El-Ela et al., 2020). This metabolism-aligned rationale is reinforced by comparable progress against other intracellular pathogens: mitochondria-targeted nanozymes that reprogram metal-dependent redox pathways, together with Ganoderma extract-capped silver nanoparticles that enter infected macrophages, suppress intracellular Salmonella Typhimurium, and recruit dendritic cells as immune adjuvants in vivo, collectively demonstrate that exploiting phase-dependent metabolic dependencies with smart nanoplatforms can markedly enhance therapeutic efficacy [52,53] (Fig. 5). Thus, the trajectory has progressed from basic formulation and targeting to synergistic antibacterial action and preliminary restoration of host metabolic equilibrium, solidifying nanotechnology as a promising direction for phase-adaptive brucellosis therapy.

Right: NIR-irradiated nanoagent potentiates cancer cell death via photothermal-enhanced, pH-responsive ion release, orchestrating a cross-regulation between ferroptosis and cuproptosis. Adapted from Ref. [52], licensed under [CC BY 4.0].

However, translating metabolism-guided nanotherapy from concept to clinically deployable interventions faces several critical challenges. One challenge lies in the complexity and spatial heterogeneity of *Brucella* metabolism in vivo, driven by microenvironmental variation between tissues and macrophage subtypes. Current metabolomic data remain largely derived from in vitro models and do not yet fully capture the metabolic landscape of persistent infection in humans. Technically, longitudinal in vivo metabolic tracing is complicated by the intracellular location of the pathogen and limited bacterial biomass.

Another major translational barrier involves the nanocarriers themselves. While many systems, including antibacterial silver nanoparticles (Ag NPs) that act via reactive oxygen species (ROS) production [55,56], demonstrate efficacy in cellular models, clinical viability requires addressing biocompatibility, immune recognition, and long-term biosafety. Recent work on mitochondria-targeted copper- and iron-based nanozyme platforms, which reprogram metal ion homeostasis to induce ferroptosis and cuproptosis while maintaining acceptable hemocompatibility in vivo, further illustrates both the therapeutic promise and the complexity of metal-based nanocarriers [52]. Metal-chelating nanosystems, for instance, must avoid dysregulating host trace-metal balance, while redox-responsive carriers must not provoke host cell cytotoxicity. Furthermore, scaling nano-formulation production to GMP-compatible, reproducible manufacturing remains a substantial barrier.

Even so, emerging research increasingly supports a workflow in which mechanistic metabolic discovery serves as the foundation for nanotherapeutic engineering. One conceptual pipeline linking these domains can be outlined as follows: (1) infection-stage metabolic profiling using multi-omics and isotopic flux tracing to identify essential metabolic nodes; (2) target prioritization based on persistence dependency and druggability; (3) design and optimization of stimuli-responsive or ligand-guided nanocarriers informed by pathogen metabolic and microenvironmental cues—a principle exemplified by the pH-responsive systems above; and (4) iterative validation in cellular, organoid, and animal models to ensure metabolic disruption translates into pathogen clearance.

Taken together, integrating mechanistic metabolic insight with nanoengineering offers a roadmap toward precision therapy for brucellosis. While significant translational barriers remain, especially around clinical validation and scalable nanomaterial development, this metabolism-nanotechnology convergence represents one of the most promising avenues for developing phase-adaptive, targeted, and potentially resistance-resilient therapies for *Brucella* infection.

## 7 Discussion

As a facultative intracellular pathogen, *Brucella* relies on precise metabolic reprogramming and tight coupling to the host milieu across acute and chronic infection. This review integrates host-level metabolic remodeling with bacterial adaptations in carbon, nitrogen, lipid, nucleotide, and metal metabolism, and highlights the role of the stringent response. At the host macroscopic level, direct plasma metabolomics in brucellosis remains limited; evidence from related models suggests that acute infection reflects inflammation/immune activation with increased glucose use and lipid rewiring [21,44]. Chronic infection more often shows signatures of organ damage, persistent inflammation, and adaptive long-term changes, including alterations in liver metabolites and amino acid/nucleotide pathways [57,58]. These shifts shape the intracellular microenvironment and drive bacterial metabolic adjustment. *Brucella*'s strategies differ by phase yet share common cores. In CCM, the acute phase requires rapid adaptation to intracellular stress and may favor readily accessible carbon (e.g., amino acids) while aligning with antioxidant defenses [48]. Chronic infection tends toward economical flux control—via regulators such as BvrR/BvrS—to sustain replication and evade immunity [2]. For nitrogen, acute infection emphasizes rapid uptake and use of abundant amino acids, whereas chronic infection adopts a more sparing program, potentially tapping alternative sources (e.g., polyamines) and interfacing with host nitrogen reprogramming [59,60].

Among other energy pathways, lipids are central: acutely, *Brucella* likely exploits host lipid pools; chronically, it refines lipid acquisition/catabolism to support long-term survival and maintain BCV integrity [45,61]. Nucleotide metabolism links to one-carbon pathways—acute infection may co-opt host one-carbon metabolism to meet high demand, whereas chronic infection relies on tighter control under limitation [46,62]. Although direct evidence is sparse, metal homeostasis is clearly critical; *Brucella* must counter nutritional immunity through phase-tailored acquisition and utilization strategies [63]. The stringent response is pivotal. Acutely, it is rapidly engaged to counter acid/oxidative/nutrient stress and pivot from growth to survival [48]. Chronically, it likely operates at low level in concert with BvrR/BvrS to maintain a "stealth" state, with potential entry into VBNC-like dormancy, as seen in Salmonella Agona [5,64]. Key gaps remain. First, comprehensive, time-resolved studies of host plasma metabolomes in brucellosis are needed to define phase-specific fingerprints; high-throughput metabolomics in clinical and animal cohorts should address this. Second, intracellular metabolic flux in *Brucella*—especially within BCVs—requires direct tracing. Stable isotope labeling (^13^C/^15^N) coupled with mass spectrometry imaging can map substrate preferences (carbon, nitrogen, lipids, nucleotides) and pathway rewiring across phases. The metal dimension is underexplored; delineating transporters and homeostatic regulators may reveal therapeutic targets. Finally, mechanistic dissection of the stringent response—(p)ppGpp synthase/hydrolase control and coordination with VirB and BvrR/BvrS—remains a priority. In sum, phase-specific metabolic strategies underlie *Brucella*'s persistence and pathogenesis. Integrating multi-omics, flux tracing, and functional genomics will clarify host-pathogen metabolic interplay and inform improved diagnostics, therapies, and vaccines for brucellosis.

To contextualize these findings within broader intracellular pathogen biology, comparative metabolic analyses offer insight into whether *Brucella* represents a conserved strategy or a metabolically divergent niche-adapted pathogen. Comparative analyses across intracellular pathogens indicate that while *Brucella*, *Mycobacterium tuberculosis*, and Salmonella share conserved metabolic themes—including metabolic flexibility, nutrient scavenging, and tight metabolism-virulence coupling—they deploy distinct strategies shaped by niche and infection kinetics [23]. Unlike Salmonella and Listeria, which retain classical glycolysis to support rapid replication in nutrient-rich cytosolic phases, *Brucella* relies on a PPP-dominant carbon metabolism and uniquely exploits erythritol, aligning with its slow-replicating vacuolar persistence strategy [8,24]. In contrast to lipid-adapted *M. tuberculosis*, which relies heavily on β-oxidation and cholesterol catabolism during granuloma persistence, *Brucella* possesses extensive amino acid transporter capacity and denitrification-linked respiration to cope with fluctuating hypoxia and nutrient scarcity [9,42]. Notably, the idea that intracellular pathogens adopt distinct metabolic specializations was first broadly conceptualized in comparative frameworks examining Salmonella, Listeria, and Mycobacterium [65,66], supporting the present conclusion that *Brucella* represents a metabolically unique yet evolutionarily conserved intracellular strategy. These distinctions position *Brucella* as a metabolically conservative yet highly specialized intracellular pathogen and emphasize that metabolism-targeted therapeutic approaches will likely require pathogen-specific tailoring rather than a unified intracellular infection model.

Despite increasing insights into *Brucella* metabolic adaptation across infection states, significant gaps remain that limit translation into effective therapeutics. Most existing evidence is derived from static omics datasets, short-term infection models, or in vitro conditions that do not fully recapitulate the metabolic heterogeneity, tissue tropism, and temporal dynamics of human infection. A major unmet need is the development of longitudinal and time-resolved metabolomics and flux-based analytical frameworks capable of capturing infection-stage-specific metabolic rewiring in vivo. Such approaches, particularly when integrated with spatial single-cell omics, isotopic tracing, and infection-model refinement, would allow a more precise identification of metabolic bottlenecks essential for persistence rather than early replication.

At the same time, the therapeutic implications of these mechanistic insights are still largely unexplored, especially regarding how metabolic vulnerabilities can be rationally leveraged for targeted nanodrug design. Future efforts should focus on building a closed-loop research framework that connects metabolic target discovery with the engineering of responsive nanocarriers, ideally capable of activating, reprogramming, or delivering therapeutics based on intracellular cues such as hypoxia, oxidative stress, nutrient limitation, and metal deprivation. Integrating nanotechnology with mechanistic metabolic understanding may enable phase-adaptive treatment strategies that selectively disrupt acute metabolic expansion or chronic persistence programs rather than applying uniform interventions.

Taken together, advancing *Brucella* research will require moving from descriptive metabolism profiling toward mechanistically informed intervention pipelines, where metabolism is not only studied as a biological signature but also harnessed as a therapeutic entry point. By combining longitudinal metabolomics, pathway-level prioritization, and smart nanotherapeutic platforms, future work may establish a foundation for precision, infection-stage-responsive therapies capable of overcoming the long-standing challenge of chronic *Brucella* persistence.

## CRediT authorship contribution statement

**Lan Yu:** Writing – review & editing, Writing – original draft, Supervision, Conceptualization. **Manyi Wen:** Writing – review & editing, Writing – original draft, Visualization. **Haitao Ding:** Supervision, Funding acquisition, Conceptualization.

## Declaration of competing interest

The authors declare that they have no known competing financial interests or personal relationships that could have appeared to influence the work reported in this paper.

## Data Availability

No data was used for the research described in the article.
